# The effects of profession‐related films on the professional pride of nursing students: A randomised controlled trial

**DOI:** 10.1002/nop2.70000

**Published:** 2024-08-20

**Authors:** Cevriye Ozdemir, Ayşe Kabuk

**Affiliations:** ^1^ Department of Medical Services and Techniques University of Kayseri Kayseri Turkey; ^2^ Department of Nursing Zonguldak Bulent Ecevit University Zonguldak Turkey

**Keywords:** films, nursing education, nursing students, professional pride

## Abstract

**Aim:**

This study aimed to determine the effects of profession‐related films on the professional pride of nursing students.

**Designs:**

The study was conducted with a randomised controlled experimental design.

**Methods:**

The sample of the study consisted of 102 students enrolled in the first year of the School of Nursing, Zonguldak Bulent Ecevit University in Türkiye in the 2022–2023 academic year. These students were randomly assigned to the experimental (*n* = 57) and control (*n* = 45) groups. The experimental group watched two documentaries and a film on YouTube with a one‐week break. Self‐administered online questionnaires were distributed via WhatsApp groups for pretests and posttests. Data were collected with a “Sociodemographic Data Form” and the “Nursing Professional Pride Scale (NPPS)” included in the questionnaire forms prepared on the Google Forms platform. Data were analysed using Spearman's rho, the Mann–Whitney *U* Test, and the Wilcoxon Signed‐Rank Test.

**Results:**

The mean age of the participants was 18.80 ± 0.99 years, 80% of the participants were female, the place where 79.4% had lived for the longest duration in their lives was the city, 85.3% had information about the profession of nursing before they started university (36.8% from the internet, 34.6% from people around them). The experimental group had significantly higher NPPS scores than the control group after the intervention (*p* = 0.017). There was also a significant increase in the dimensions of professional feeling (*p* = 0.012) and desire to continue the profession in the experimental group (*p* = 0.002).

**Patient or Public Contribution:**

Patients and public were not involved in this research.

## 1 INTRODUCTION

The profession of nursing has professional values, principles, and standards. Nursing care is a holistic and enduring approach (Stecker, 2016). Therefore, nurses are in more contact with patients or healthy individuals than other health professionals (Delaney & Bark, 2019; Evén et al., 2019). This is a good time for nurses to explain nursing to patients and society. In addition to nursing knowledge and skills, pride in nursing is also important to present the profession of nursing in the desired manner. Nurses with high professional pride will also increase the positive perceptions of nursing in society. The perception of nursing in society supports the perception of the professional pride of nurses (Delaney & Bark, 2019; Evén et al., 2019; İlaslan et al., 2021; Vikström & Johansson, 2019). The perception of nursing in society also influences the decision of individuals to attend nursing schools. Therefore, nurses who eagerly choose to pursue a career in nursing and identify with and adhere to their profession are likely to develop a stronger professional identity and perceive their work more positively (Alharbi et al., 2020; Guo et al., 2017).

The supportive position of nurses in providing comprehensive care, protection, and health maintenance through their holistic approach to assessing the needs of healthy individuals, patients, and patient families helps build positive perceptions of their professional pride (Obeidat et al., 2018; Vikström & Johansson, 2019). In addition to these strengthening factors, some social, environmental, and personal factors that reduce the motivation or satisfaction of nurses affect their professional pride. These factors include a hierarchical work environment that disregards physical and mental health, irrelevant tasks outside of job descriptions, a negative public image of nursing, low wages, insufficient autonomy, medication‐centred care, and other personal experiences (Obeidat et al., 2018; Valizadeh et al., 2018). Nursing students should be prepared for the positive and negative situations they may encounter in their professional lives.

Previous studies have addressed the factors that affect professional life and pride. Tjoflåt et al. found that nurses take pride in their work, are motivated to help those in need, and are satisfied with patient‐centred care (Tjoflåt et al., 2018). Higher professional pride and job satisfaction have been reported to result in a stronger intention to stay at one's job (Sneltvedt & Sørlie, 2012). The same study revealed that newly graduated nurses are proud of and highly committed to their job and want to do it in the best possible way. Nurses with professional pride and motivation have a positive attitude towards their jobs. On the other hand, those with low professional pride and motivation experience more physical and mental health problems (e.g., feelings of inadequacy, worthlessness, depression, burnout, low job satisfaction, lack of motivation) and stronger intentions to turnover (Kim et al., 2019; Vikström & Johansson, 2019). Job dissatisfaction negatively affects the mental health of nurses and the quality of the care they provide, leading to poor patient health outcomes (Obeidat et al., 2018; Tjoflåt et al., 2018; Valizadeh et al., 2018). Therefore, it is paramount to ensure that nurses take pride in their work and are sufficiently motivated and satisfied to provide high‐quality care.

In today's education systems, advanced communication technologies and diverse learning methods are preferred for effective learning. Active learning methods should be used in nursing education, which requires cognitive, affective, and psychomotor gains.

Films are recognised as practical educational resources to encourage the retention of knowledge and the development of desired behaviours (Tjoflåt et al., 2018). Films can trigger student discussions and reinforce a lesson, thus encouraging practice far beyond the written course material. Certain moments in a film can activate the brain's visual areas, making abstract concepts easier to understand (Oh et al., 2012). Watching and thinking about films can involve students in their learning and enable them to understand complex concepts more in depth (Arveklev et al., 2018). Films in education programs in health‐related professions are becoming more and more prominent in the literature (Arveklev et al., 2018; Oh et al., 2012). They are also widely used in nursing education and training. However, the numbers of YouTube videos, films, and other resources specific to the field of nursing are increasing only slowly. Additionally, it is important that films and other resources related to nursing are related to both education and healthcare. This allows nursing professionals to incorporate approved educational technologies such as videos to promote lasting and continuous education. In the existing literature, no study has used YouTube, films, and similar resources specific to the professional pride of nurses.

Because films provide a means to partake in a situation indirectly, nurses and nursing students can experience a situation realistically and feel it from the patient's point of view. Although there are instructional uses of films, television, and videos in the literature, not much is known about the use of films in nursing education. Therefore, this study aims to determine the effects of profession‐related films on the professional pride of nursing students.

## 2 METHODS

### 2.1 Hypothesis

H_1_: Nursing students who watch profession‐related films have higher levels of professional pride than those who do not.

### 2.2 Design

**FIGURE 1:**
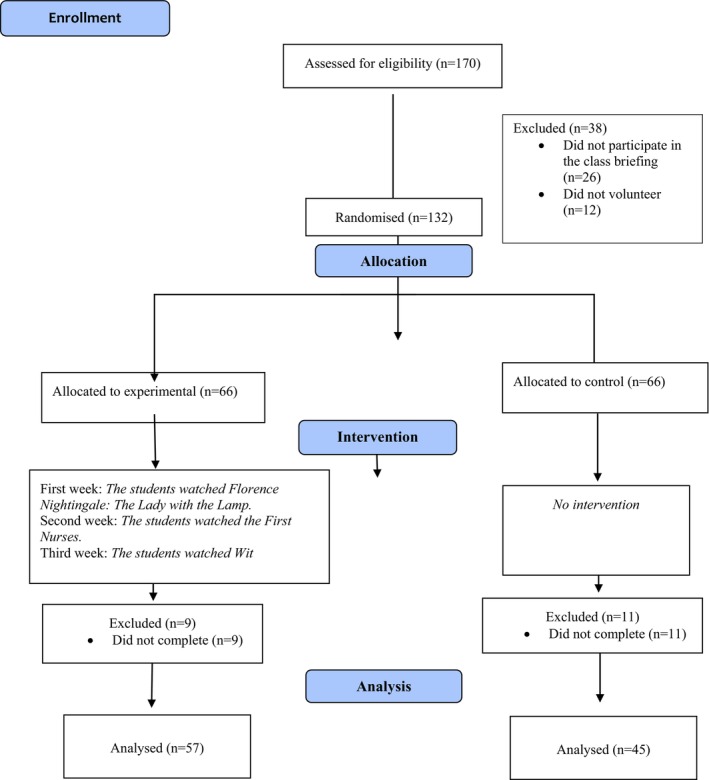
CONSORT 2010 flow diagram.

This study aimed to determine the effects of profession‐related films on the professional pride of nursing students. The study was conducted with a randomised controlled experimental design and complied with the Consolidated Standards of Reporting Trials (CONSORT) Checklist (Figure 1). The Clinical Trials registration number of the study was received as NCT05675839.

### 2.3 Participants and sample size calculation

The sample of our study consisted of first‐year nursing students. All first‐year nursing students are enrolled in the Physiology, Psychology, Microbiology‐Parasitology, Interpersonal Relations in Nursing, Anatomy, Histology, and Introduction to Nursing courses in the first semester. Among these courses is the Introduction to Nursing course, which introduces the profession of nursing as its name suggests. Within the scope of the Introduction to Nursing course, the roles, ethics, and values of nursing are explained theoretically in only one class hour a week. However, explaining nursing theoretically for one class hour a week in this course is complicated. Therefore, it is thought that active learning methods should be used. The population of the study included 170 first‐year students enrolled in the nursing department at Zonguldak Bulent Ecevit University, Türkiye, in the 2022–2023 academic year.

The sample size required to conduct the study was calculated using the G*Power 3.1.9.7 program. The sample size was calculated for an independent‐samples *t*‐test as a minimum of 102 by taking an effect side of 0.50 (*d* = 0.50), a 5% margin of error (*α* = 0.05), and 80% power (1‐β = 0.80) (Cohen, 2013; Faul et al., 2007). The study was started by inviting 170 students enrolled in the first year of the program. Twenty‐six of the invited students did not attend the first meeting held for the study, and 12 students did not volunteer to participate. The 132 students who participated in the study were divided into the experimental (*n* = 66) and control (*n* = 66) groups, and they filled out the data collection forms. Nine students in the experimental group and 11 in the control group did not complete the study. The study was completed with 57 students in the experimental group and 45 students in the control group (Figure 1).

### 2.4 Inclusion and exclusion criteria

The inclusion criteria were being enrolled in the first year of the nursing program for the first time in the 2022–2023 academic year, not repeating the class, and volunteering to participate in the study. The exclusion criteria were not being a first‐year student, having been a nursing student in another school before, and not agreeing to participate in the study.

### 2.5 Outcome measures

The data were collected using a Sociodemographic Data Form and the Nursing Professional Pride Scale.

#### 2.5.1 Sociodemographic data form

The form was prepared by the researchers to collect the sociodemographic data of the participants such as their age, gender, place of residence, and their thoughts about the profession of nursing (İlaslan et al., 2021; Stecker, 2016; Vikström & Johansson, 2019).

#### 2.5.2 Nursing professional pride scale (NPPS)

The scale was developed by JaeHee et al. to measure the pride of nurses regarding their profession (Jeon et al., 2020). The Turkish validity and reliability study of the scale was conducted by Aydin et al (Aydin et al., 2022). According to the study, the Turkish version of NPPS had a content validity index of 0.95 and a Cronbach's alpha coefficient of 0.89, and a five‐factor structure with acceptable psychometric properties (*χ* ^2^/df = 2.85, RMSEA = 0.079, CFI = 0.92, SRMR = 0.08) (Aydin et al., 2022).

The scale includes 27 items and five subscales. Items are scored on a five‐point Likert‐type scale (1 = strongly disagree, 2 = disagree, 3 = undecided, 4 = agree and 5 = strongly agree). The subscales include feeling of vocation (six items, 1, 2, 3, 4, 5 and 6), role satisfaction (six items, 7, 8, 9, 10, 11 and 12), role of problem solver (six items, 13, 14, 15, 16, 17, and 18), self‐achievement (four items, 19, 20, 21, and 22), and willingness to stay (five items, 23, 24, 25, 26 and 27). The total score ranges from 27 to 135, where higher scores indicate more professional pride (Aydin et al., 2022; Jeon et al., 2020). In our study, the Cronbach's alpha coefficient of NPPS was 0.891 in the pretest (subscale coefficients in the range of 0595–0.818) and 0.948 in the posttest (subscale coefficients in the range of 0.839–0.902).

### 2.6 Data collection

In the first semester of their first year, nursing students are enrolled mainly in basic medical sciences courses. There is no clinical practice in this period. Only theoretical information about the professionalism, roles, ethical principles, and values of nursing is given in a one‐hour course called Introduction to Nursing every week. The participants were gathered in a classroom at a defined time and informed about the study, and their questions were answered. After obtaining their written consent, the participants filled out the Sociodemographic Data Form and NPPS in the pretest. The data collection forms were sent to the participants with the URL of the Google Forms document via a WhatsApp group. The pretest forms were filled in before the participants started any courses. The participants had not taken any nursing‐related courses when the forms were filled in.

The participants in the experimental group watched a film and two documentaries. No intervention was applied to the control group. During the practices, all participants took part in the ‘Introduction of the Course’ for 1 h, ‘History of Nursing’ for 1 h, and ‘Professionalisation and Nursing’ for 1 h as topics within the scope of the ‘Introduction to Nursing’ course. The topics were explained only theoretically. Three weeks later, all participants completed the posttest NPPS measurements.

### 2.7 Interventions

#### 2.7.1 Randomisation

A computer‐generated (www.randomizer.org/) sequence of random numbers was used to randomise the participants into either group.

Before the interventions, the film and the documentaries were selected. Ten experts with PhD degrees in the Fundamentals of Nursing were consulted for this process. The purpose of the study was explained to the experts, who were asked to suggest five films and five documentaries that deal with the professional aspects of nursing, which were available on YouTube. Based on the suggestions of the experts, two documentaries (First Nurses, 2018; Nightingale, 2019) and a film (Wit, 2001) were selected with a joint decision.

The participants in the experimental group were asked to watch Florence Nightingale: The Lady with the Lamp first. After a week had passed, they were asked to watch First Nurses. Finally, after another week had passed, they were asked to watch the film Wit. The participants were asked to share photos during the watching of these videos to encourage each other, and small gifts such as coffee and chocolate were offered to the participants. The participants took part in social interactions and motivated each other to watch the videos by sending the photos they took during the film watching moments to the WhatsApp group created by the researchers to conduct the study.

The films used in the study.

*Florence Nightingale: The Lady with the Lamp* (Documentary) is about Florence Nightingale. She was born into a life of wealth and privilege, in an age when women were expected to simply marry, have children, and not do much else. Nightingale dedicated her life to helping the sick unheard of in Victorian England. In the Crimean War, she saved countless lives and came back a hero, sought after by royalty and governments around the world. Florence Nightingale dedicated her life to establishing the modern profession of nursing. The documentary is 28 min long and was released in 2019. (https://www.youtube.com/watch?v=yWs0tlYRpEc&t=1472s) (Nightingale, 2019).

*The First Nurses* (Documentary) is about the life of Safiye Huseyin Elbi (1881–1964), known as one of the founders of modern nursing. Florence Nightingale was her role model. The documentary is 27 min long and was released in 2018. It is about Mrs. Elbi, who, after graduation, served as a nurse in the Battle of Gallipoli and received multiple medals for her achievements while continuing her contributions to nursing education (https://www.youtube.com/watch?v=XsabHOiF0CY&t=286s) (First Nurses, 2018).

*Wit* (Film) is about the life story of a woman diagnosed with cancer, her difficulties during treatment, and her communication with the nurse (First Nurses, 2018). The film was released in 2001, and it is 1 h and 35 min long. The film's main character is Vivian Bearing, played by Emma Thompson. A professor of English Literature, Bearing is an accomplished academic specialising in the poetry of the 17th‐century English poet John Donne (1572–1631). She consults the doctor because of the pain in her stomach and learns that she has ovarian cancer. Discovering that Vivian Bearing's cancer is at an advanced stage, the physician recommends an aggressive dose of chemotherapy for her treatment and includes it in her scientific work. Resolved at the start of therapy, Bearing starts to lose her will over time as she experiences the adverse effects of chemotherapy and begins to suffer visibly both in the physical and emotional sense, but the physician does not interrupt the treatment protocol. Nurse Susie Monahan is the primary nurse responsible for Bearing. Monahan provides nursing care with respect and understanding during her illness and advocates for Bearing when necessary (https://www.youtube.com/watch?v=2s4ozvI_hzY&t=141s) (Wit, 2001).

### 2.8 Analysis

The IBM SPSS Statistics version 23 (IBM Inc., Armonk, NY, USA) was used for data analysis. The nominal variables are presented with frequency and percentage values, while the ordinal variables are presented with mean and standard deviation values. The data showed a non‐normal distribution according to the Shapiro–Wilk test results. For this reason, chi‐squared, Spearman's rho, the Mann–Whitney *U* Test, and the Wilcoxon Signed‐Rank test were used to compare the mean scale scores of the participants. The results were evaluated in a 95% confidence interval and at a significance level of *p* < 0.05.

### 2.9 Ethical considerations

Ethical approval was obtained from the Ethics Committee of Kayseri University (Date and No:13/09/2022 and E.36946). Permission was obtained from the institution where the study was conducted. Written consent was obtained from each participant.

## 3 RESULTS

The mean age of the participants was 18.80 ± 0.99 years, 79.4% of them were female, the place where 85.3% had lived for the longest time was the city centre, and 66.7% were currently living in dormitories. It was stated by 68.6% of the participants that their income was sufficient, 68.6% willingly chose to become a nurse, 53.9% had nurses in their social environment, 79.4% had obtained information about the profession of nursing from the internet (36.8%) or the people around them (34.6%) before attending university. It was learned that 51.0% of the participants had negative views about the profession of nursing before university, 73.5% said they were planning to as nurses in the future, and 79.4% stated they were not concerned about unemployment. The groups did not significantly differ in terms of their descriptive characteristics (*p* > 0.05) (Table 1).

**TABLE 1:** Descriptive characteristics (*n* = 102).

Descriptive characteristics	Total		Experimental		Control		*p*‐Value^a^
	Mean ± SD (min–max)		Mean ± SD (min–max)		Mean ± SD (min–max)		
Age (years)	18.80 ± 0.99 (17–22)		18.89 ± 1.10 (17–22)		18.69 ± 0.82 (17–20)		0.384
	*n*	%	*n*	%	*n*	%	*p*‐Value^b^
Gender							
Female	81	79.4	45	79.0	36	80.0	0.896
Male	21	20.6	12	21.0	9	20.0	
Place of residence for longest time							
City	87	85.3	51	89.5	36	80.0	0.06
Town	5	4.9	‐	‐	5	11.1	
Village	10	9.8	6	10.5	4	8.9	
Current place of living							
Dormitory	68	66.7	39	68.4	29	64.5	0.914
Home with Friends	26	25.5	16	28.1	10	22.2	
Home with Family	8	7.8	2	3,5	6	13.3	
Income level							
Low	20	19.6	11	19.3	9	20.0	0.227
Medium	70	68.6	42	73.7	28	62.2	
High	12	11.8	4	7.0	8	17.8	
Willingly chose to become a nurse							
Yes	70	68.6	40	70.2	30	66.7	0.705
No	32	31.4	17	29.8	15	33.3	
Knows nurses in their social environment							
Yes	55	53.9	27	47.4	28	62.2	0.135
No	47	46.1	30	52.6	17	37.8	
Had information about the profession of nursing before university							
Yes	81	79.4	44	77.2	37	82.2	0.533
No	21	20.6	13	22.8	8	17.8	
Source of information about the profession of nursing obtained before university (multiple choices were allowed)							
Internet	68	36.8	36	35.3	32	38.1	0.255
Environment	64	34.6	34	33.3	30	35.7	
Nurses	37	20.0	23	22.5	15	17.9	
Relatives	10	5.4	5	5.0	5	6.0	
Other	6	3.2	4	3.9	2	2.3	
General view of the profession of nursing before coming to nursing school							
Positive	37	36.3	23	40.4	14	31.2	0.934
Undecided	13	12.7	3	5.2	10	22.2	
Negative	52	51.0	31	54.4	21	46.6	
Planning to work as a nurse in the future							
Yes	75	73.5	39	68.4	36	80.0	0.199
Undecided	24	23.5	16	28.1	8	17.8	
No	3	2.9	2	3,5	1	2.2	
May think of changing one's department							
Yes	4	3.9	2	3,5	2	4.4	0.944
Undecided	15	14.7	8	14.0	7	15.6	
No	83	81.4	47	82.5	36	80.0	
Has concerns about future unemployment							
Yes	21	20.6	13	22.8	8	17.8	0.706
No	81	79.4	44	77.2	37	82.2	
Total	102	100	57	100	45	100	

^a^ Spearman's rho correlation analysis.

^b^ Chi‐squared test.

Before the intervention, the mean total NPPS scores of the participants was 98.31 ± 17.37 in the experimental group and 99.42 ± 13.48 in the control group, with no significant difference between them (*p* = 0.504). After the intervention, the mean total NPPS total score of the participants was 106.28 ± 17.57 in the experimental group and 95.08 ± 24.30 in the control group, demonstrating a significant difference between the groups (*p* = 0.017), where the experimental group had a higher mean total NPPS score than the control group (Table 2).

**TABLE 2:** Comparison of nursing professional pride scale total and dimension scores (*n* = 102).

Scale dimensions	Experimental group	Control group	Test value	*p*‐Value^a^
	Mean ± SD	Mean ± SD		
Feeling of vocation				
Pretest	21.64 ± 0.58	21.31 ± 0.50	*U* = 1095.5	0.205
Posttest	23.47 ± 0.58	21.17 ± 0.85	*U* = 974.0	0.037*
*Test value*	*z* = −2.508	*z* = −0.175		
*p*‐value^b^	0.012*	0.861		
Role satisfaction				
Pretest	21.94 ± 0.59	19.28 ± 0.52	*U* = 951.5	0.025*
Posttest	23.33 ± 059	19.28 ± 0.76	*U* = 899.0	0.009*
*Test value*	*z* = −1.941	*z* = −0.217		
*p*‐value^b^	0.052	0.828		
Role of problem solver				
Pretest	22.50 ± 0.57	23.35 ± 0.60	*U* = 1214.5	0.641
Posttest	24.21 ± 0.56	21.84 ± 0.89	*U* = 1004.0	0.058
*Test value*	*z* = −1.889	*z* = −1.512		
*p*‐value^b^	0.059	0.130		
Self‐achievement				
Pretest	15.24 ± 0.41	15.66 ± 0.42	*U* = 1245.0	0.797
Posttest	16.24 ± 0.37	14.42 ± 0.63	*U* = 955.50	0.025*
*Test value*	*z* = −1.826	*z* = −1.809		
*p*‐value^b^	0.068	0.070		
Willingness to continue the profession				
Pretest	18.57 ± 0.46	19.80 ± 0.45	*U* = 1065.0	0.138
Posttest	20.40 ± 0.47	18.35 ± 0.78	*U* = 1012.0	0.062
*Test value*	*z* = −3.105	*z* = −1.689		
*p*‐value^b^	0.002*	0.091		
Total				
Pretest	98.31 ± 17.37	99.42 ± 13.48	*U* = 1183	0.504
Posttest	106.28 ± 17.57	95.08 ± 24.30	*U* = 930	0.017*
*Test value*	*z* = −2.536	*z* = −0.677		
	0.011*	0.499		

^a^ Mann–Whitney *U* test.

^b^ Wilcoxon Signed‐Rank test.

* *p* < 0.05.

In the NPPS subscales, the experimental group had higher mean posttest scores of feeling of vocation (*p* = 0.037), role satisfaction (*p* = 0.009), and self‐achievement (*p* = 0.025) in comparison to the control group (*p* < 0.05). On the other hand, there was no significant difference between the experimental and control groups in terms of their willingness to stay subscale scores (*p* = 0.062) (Table 2).

The comparison of the pretest and posttest NPPS total scores of the participants in the experimental group demonstrated a significant increase (*p* = 0.011). There were also statistically significant differences in their scores in the feeling of vocation (*p* = 0.012) and willingness to stay (*p* = 0.002) subscales (Table 2).

The pretest‐posttest comparisons of the participants in the control group demonstrated no statistically significant difference in terms of their NPPS total or subscale scores (*p* > 0.05) (Table 2).

The NPSS scores of the participants were compared with reference to their descriptive characteristics. In the experimental group, the mean NPPS scores of the female participants (*p* = 0.008), the participants whose place of residence for the longest duration in their lives was the city (*p* = 0.006), those who stayed in dormitories (*p* = 0.018), those who had equivalent income and expense levels (*p* = 0.006), and those who knew a nurse (*p* = 0.045) had significantly higher scores. Additionally, the mean NPPS score of those in the experimental group with positive or negative views about nursing before coming to school increased significantly from the pretest to the posttest (*p* = 0.002), whereas no significant change was observed in the scores of those who were undecided. Again, in the experimental group, the mean NPPS score of the participants who were not concerned about unemployment increased (*p* = 0.042). In the control group, the mean NPPS score of the participants who were undecided about changing their department decreased significantly (*p* = 0.043). For both the experimental and control groups, there was no significant difference in the mean NPSS scores of the participants based on their other descriptive characteristics (*p* > 0.05) (Table 3.).

**TABLE 3:** Comparison and mean NPPS scores based on descriptive characteristics (*n* = 102).

Descriptive features	Total		Experimental		Test value, *p*‐value^a^	Control		*Test value*, *p*‐value^a^
			Pretest	Posttest		Pretest	Posttest	
	Mean ± SD		Mean ± SD	Mean ± SD		Mean ± SD	Mean ± SD	
Age (years)	18.80 ± 0.99 (17–22)		98.31 ± 17.37	106.28 ± 17.57	*z* = −2.536, *p* = 0.011^*^	99.42 ± 13.48	95.08 ± 24.30	*z* = −0.677, *p* = 0.499^a^
	*n*	%						
Gender								
Female	81	79.4	99.31 ± 16.77	108.84 ± 13.31	*z* = −2.640 *p* = 0.008^*^	90.02 ± 14.58	93.45 ± 26.27	*z* = −0.274 *p* = 0.784
Male	21	20.6	94.58 ± 19.81	96.66 ± 27.12	*z* = −0.471 *p* = 0.638	101.25 ± 6.67	102.62 ± 9.07	*z* = −1.836 *p* = 0.066
Place of residence for the longest time								
City	87	85.3	94.94 ± 18.05	106.98 ± 18.06	*z* = −2.737, *p* = 0.006^*^	100.75 ± 13.43	98.52 ± 21.16	*z* = 0.0, *p* = 1.000
Town	5	4.9	–	–	–	95.80 ± 8.46	78.20 ± 29.29	*z* = −0.355, *p* = 0.176
Village	10	9.8	101.50 ± 10.40	100.33 ± 12.16	*z* = −0.105, *p* = 0.917	92.00 ± 18.67	85.25 ± 39.30	*z* = −0.535, *p* = 0.593
Current place of living								
Dormitory	68	66.7	96.53 ± 20.43	107.56 ± 14.48	*z* = −2.373, *p* = 0.018^*^	100.51 ± 14.22	93.13 ± 26.26	*z* = −1.431, *p* = 0.153
Home with friends	26	25.5	94.00 ± 1.41	96.00 ± 16.97	*z* = −0.447, *p* = 0.655	101.16 ± 10.98	92.83 ± 20.06	*z* = −0.734, *p* = 0.463
Home with family	8	7.8	103.18 ± 5.83	104.43 ± 24.09	*z* = −0.937, *p* = 0.349	95.20 ± 12.93	102.10 ± 21.13	*z* = −1.581, *p* = 0.114
Income level								
Low	20	19.6	102.90 ± 9.62	98.18 ± 26.50	*z* = −0.311, *p* = 0.755	90.66 ± 16.99	92.55 ± 33.67	*z* = −0.280, *p* = 0.779
Medium	70	68.6	97.26 ± 19.49	108.19 ± 14.98	*z* = −2.763, *p* = 0.006^*^	100.87 ± 11.07	95.32 ± 22.33	*z* = −1.974, *p* = 0.330
High	12	11.8	96.75 ± 6.34	108.50 ± 7.68	*z* = −1.461, *p* = 0.144	104.25 ± 14.38	97.12 ± 21.89	*z* = −0.420, *p* = 0.674
Willingly chose to become a nurse								
Yes	70	68.6	100.62 ± 17.34	108.05 ± 18.13	*z* = −1.923, *p* = 0.054	99.20 ± 15.33	93.06 ± 26.99	*z* = −1.014, *p* = 0.311
No	32	31.4	92.88 ± 16.70	102.11 ± 15.91	*z* = −1.610, *p* = 0.107	99.86 ± 9.17	99.13 ± 17.88	*z* = −0.426, *p* = 0.670
Knows nurses in their social environment								
Yes	55	53.9	101.37 ± 9.92	108.70 ± 15.69	*z* = −2.007, *p* = 0.045^*^	101.00 ± 11.87	100.07 ± 18.68	*z* = −0.108, *p* = 0.914
No	47	46.1	95.56 ± 21.87	104.10 ± 19.11	*z* = −1.786, *p* = 0.074	96.82 ± 15.83	86.88 ± 30.33	*z* = −1.397, *p* = 0.162
Had information about the profession of nursing before university								
Yes	81	79.4	97.63 ± 16.20	108.20 ± 14.56	*z* = −3.147, *p* = 0.002^*^	99.59 ± 14.79	93.89 ± 26.53	*z* = −1.016, *p* = 0.310
No	21	20.6	100.61 ± 21.48	99.76 ± 24.93	*z* = −0.589, *p* = 0.556	98.62 ± 4.10	100.62 ± 7.00	*z* = −0.634, *p* = 0.526
General view of the profession of nursing before coming to nursing school								
Positive	37	36.3	101.13 ± 16.79	110.91 ± 16.76	*z* = −2.209, *p* = 0.027^*^	104.50 ± 14.88	90.28 ± 31.62	*z* = −1.610, *p* = 0.107
Undecided	13	12.7	98.00 ± 10.81	93.66 ± 16.74	*z* = −0.535, *p* = 0.593	99.60 ± 9.15	95.70 ± 15.75	*z* = −0.593, *p* = 0.553
Negative	52	51.0	96.25 ± 18.40	104.06 ± 17.73	*z* = −1.813, *p* = 0.070	95.95 ± 13.70	98.00 ± 22.62	*z* = −0.730, *p* = 0.435
Planning to work as a nurse in the future								
Yes	75	73.5	99.71 ± 17.77	107.89 ± 14.51	*z* = −1.887, *p* = 0.059	99.83 ± 14.53	94.14 ± 24.68	*z* = −1.257, *p* = 0.209
Undecided	24	23.5	95.50 ± 0.70	93.50 ± 13.42	*z* = −1.603, *p* = 0.109	111.00 ± 0.0	118.00 ± 0.0	*z* = −0.632, *p* = 0.528
No	3	2.9	95.25 ± 17.63	103.93 ± 23.94	*z* = −0.447, *p* = 0.655	96.12 ± 7.37	96.25 ± 24.15	*z* = 1.0, *p* = 1.00
May think of changing one's department								
Yes	4	3.9	94.50 ± 2.12	105.50 ± 3.53	*z* = −1.342, *p* = 0.180	100.50 ± 14.84	81.00 ± 52.32	*z* = −0.447, *p* = 0.655
Undecided	15	14.7	98.65 ± 18.78	108.36 ± 14.62	*z* = −1.480, *p* = 0.139	101.52 ± 12.65	94.11 ± 24.43	*z* = −2.028, *p* = 0.043^*^
No	83	81.4	97.25 ± 9.52	94.25 ± 29.48	*z* = −2.485, *p* = 0.051	88.28 ± 13.80	104.14 ± 15.00	*z* = −0.589, *p* = 0.556
Has concerns about future unemployment								
Yes	21	20.6	93.53 ± 20.29	102.61 ± 25.74	*z* = −1.490, *p* = 0.136	93.62 ± 13.82	89.37 ± 33.86	*z* = −0.280, *p* = 0.779
No	81	79.4	99.72 ± 16.41	107.36 ± 14.56	*z* = −2.030, *p* = 0.042^*^	100.67 ± 13.26	96.32 ± 22.13	*z* = −0.893, *p* = 0.372

^a^ Wilcoxon Signed‐Rank test.

* *p* < 0.05.

## 4 DISCUSSION

Nurses are introduced to real working conditions while they are still students in laboratory and clinical practice. In this process, they learn the norms, values, behaviours, attitudes, and culture of the profession to which they want to belong. Every contact with the profession of nursing aims to help nursing students develop a professional identity among future nurses (Arreciado Marañón & Isla Pera, 2015).

In the context of education programs in health‐related professions, films are increasingly prominent in the literature (McKie, 2012). They are also widely used in nursing education and training. Additionally, the numbers of YouTube videos directly pertaining to the field of nursing are increasing. Approved and available educational technologies such as videos support continuity in education (Mellor et al., 2013).

According to the results of our study, in which we investigated the effects of films about the profession of nursing on pride in nursing students, after the intervention, the experimental group was found to have a significantly higher mean total NPPS score than the control group (Table 1), indicating that the films about the profession that the participants watched had an impact in increasing their professional pride levels. Films can stir emotions and feelings and affect the viewer's knowledge, attitudes, beliefs, and behaviour. According to current research, using clips or films prompts self‐reflection and encourages discussion about positive health behaviours. In this context, films can provide real‐life descriptions of subjects that are difficult to repeat in the classroom, and they can convey the message to the student much more clearly and give a humane element that can reinforce the knowledge of reality (Herman et al., 2014). In a study investigating the effects of having nursing students watch videos, students stated that watching and discussing TV series in which nurses played critical roles rose a sense of pride in their chosen profession (McAllister et al., 2015). Given that nurses are essential for the health of the world's population, it is imperative that standards of nursing education are improved globally (Mason et al., 2016; Turale, 2015).

After the intervention, the experimental group had higher scores than the control group in the feeling of vocation, role satisfaction, role of problem solver, and self‐achievement subscales of NPPS (Table 2). The use of films in education provides benefits in terms of triggering emotional reactions, creating an environment for discussion, improving clinical decision‐making, and providing indirect learning (Park & Cho, 2021). The use of film clips in the classroom draws attention to changes in the attention span of students and is reported to provide learning stimuli that meet the participant's needs. Thought‐provoking, candid, and realistic video scenes can encourage emotional responses and commentary on social issues and reinforce the caring elements of nursing roles (de Braganca & Nirmala, 2018). This people‐centred approach to experiential learning enables nursing students to learn through film clips and discussions, where individual differences can be accepted, and problems can be addressed in a controlled environment. It provides a mechanism for understanding situations such as professional pride and role satisfaction, which are difficult to describe in the classroom but essential to nursing education.

The pretest‐posttest comparison of the scores of the experimental group demonstrated a significant increase in their NPPS total, feeling of vocation subscale, and willingness to stay subscale scores (Table 2). The results of the study showed that films about the profession watched by the participants had a desirable effect, enhancing their feelings about their vocation and willingness to stay in the profession. It has been reported that films help students develop various experiences, including emotions, feelings, knowledge, actions, skills, and attitudes (de Braganca & Nirmala, 2018; Doğan & Şendir, 2022; Jefferies et al., 2021; Kalra, 2013; Mason et al., 2016; McAllister et al., 2015; Oh & Steefel, 2016; Park & Cho, 2021; Turale, 2015; Zeppegno et al., 2015). Furthermore, using films in education also improves components related to professional standing, such as understanding the patient experience, developing communication skills, and increasing self‐reflection (Jeon et al., 2020). In other words, using films in education activates the brain's visual areas, allowing students to experience a given situation, empathise emotionally, and formulate solutions while actively participating in the class and discussing solutions.

Among the participants in the experimental group, the pretest and posttest NPPS mean scores of those who were female, those who lived in the city for the longest time in their lives, those who lived in dormitories, and those knew a nurse in their personal lives were found to be significantly different, and their posttest scores were higher. In the literature review, the results of previous studies were found to support our findings (Abhicharttibutra et al., 2017; Sinclair et al., 2016). It is thought that female students have a more positive approach to the video learning method than male students. In this context, their mean NPPS scores are higher (Sinclair et al., 2016). It was considered that since the place where most of the participants in this study lived for the longest time in their lives was the city and most were staying in dormitories, the changes in their pretest posttest NPPS scores were significant. Additionally, was thought that city life allows a better observation of the profession and that most students living in dormitories may have lower or moderate levels of income, which may have led them to embrace the profession for which they were studying more. The finding that the mean scores of the participants who knew nurses in their personal lives were significantly higher may be related to their more positive attitudes towards the profession based on their observation of people practicing the profession closely. It has been reported that using films in the education of nursing students increases their knowledge and understanding, makes them more self‐confident and raises their motivation. In this context, it is seen that the intervention in the study positively affected the views of the participants about the profession of nursing. A well‐educated and adequate nursing workforce improves public health and the standards of nursing care. Thus, analysing nursing workforce policies will help nurses learn from the past and develop better policies for the future (Abhicharttibutra et al., 2017). Nurses are critical to health policy. Therefore, improving nursing education will be helpful in the successful reduction of shortages and ultimate improvement of public health through nursing practice. In this study, it was found that showing films related to the profession of nursing to nursing students contributed positively to their professional pride (Arkan & Bostanli, 2024). Therefore, the intervention in this study may be an effective strategy to develop professional pride and associated factors in nursing students. Nursing education reform influences health policies as an important strategy for improving the performance of the health workforce and thus the functioning of health systems.

### 4.1 Limitations

The strength of this study is that it is registered on clinicaltrial.gov, it has a randomised and controlled experimental design, and the researchers spent considerable time during the interventions.

This study also had some limitations that should be addressed. First, the reliance on self‐reported measures has the potential for response and measurement errors. In the study, films and documentaries were shown to the experimental group. No additional intervention was performed in the control group. However, students in both groups continued to take the ‘Introduction to Nursing’ course. This course was provided in one class hour a week, and brief information about the history of nursing was given. This may have been impressive, but since the same information was given to both groups, it was not expected to affect the results. Finally, the students needed internet connection and devices such as computers/phones. Even though they used the university's facilities, some students could not complete the intervention because they could not access the internet. It is not possible to generalise the results obtained because the study was conducted in a single school and in a certain period of time.

## 5 CONCLUSIONS

In summary, the most notable result of this study was the importance of supporting student satisfaction with effective teaching methods to train nursing students with a strong sense of pride and competence in nursing knowledge and skills. In particular, the use of resources such as YouTube, films, and other similar materials specific to the field of nursing will be more effective than other audiovisual tools. If these issues are addressed early in the education programs of nurses, the future success of students and their possibility of progression in nursing may increase. We propose an innovative approach to having a specific vision for nursing and being proud of everything we have achieved in the profession.

## FUNDING INFORMATION

No external or intramural funding was received.

## CONFLICT OF INTEREST STATEMENT

The authors have no conflict of interest to disclose.

## ETHICS STATEMENT

The ethics committee's approval was obtained from The Ethics Committee of Kayseri University (Date and No: 13.09.2022 and E.36946).

## CLINICAL TRAIN REGISTRATION

Clinical Trials registration number was received as NCT05675839.

## Data Availability

The data that support the findings of this study are available on request from the corresponding author. The data are not publicly available due to privacy or ethical restrictions.
